# Early transcriptomic perturbations highlight the spinal cord as a key pathogenic region in spinocerebellar ataxia type 3

**DOI:** 10.3389/fncel.2025.1735225

**Published:** 2026-01-14

**Authors:** Jacen Emerson, Brianna S. Nelthrope, Emma A. Walker, Grace Mao, Hannah K. Shorrock, Hayley S. McLoughlin

**Affiliations:** 1 Department of Neurology, University of Michigan, Ann Arbor, MI, United States; 2 Neuroscience Graduate Program, University of Michigan, Ann Arbor, MI, United States; 3 The RNA Institute and Department of Biological Sciences, College of Arts & Sciences, University at Albany-SUNY, Albany, NY, United States; 4 Department of Human Genetics, University of Michigan, Ann Arbor, MI, United States

**Keywords:** alternative splicing, inflammation, oligodendrocyte, polyglutamine (polyQ) disease, SCA3, spinal cord, spinocerebellar ataxia

## Abstract

Spinocerebellar ataxia type 3 (SCA3) is a neurodegenerative disease caused by polyglutamine repeat expansion in the *ATXN3* gene. Despite the ubiquitous expression of ATXN3 throughout the body, SCA3 pathology is most pronounced in select, vulnerable central nervous system regions. Notably, spinal cord atrophy that is detectable by MRI emerges prior to ataxia symptom onset and progresses with disease severity. However, the pathogenic molecular signatures of the SCA3 spinal cord remain largely unexplored. Here, we present the first comprehensive analysis of the spinal cord transcriptome in SCA3 using both human and mouse model tissue. Our data reveal both early and progressive transcriptional dysregulation in the spinal cord, impacting key biological processes such as lipid metabolism, inflammation, cellular structure, and nucleic acid processing. Transcriptomic profiling of *Atxn3* knockout mouse spinal cord revealed only subtle transcriptional changes with little overlap to those in SCA3 knock-in mice, indicating that spinal cord pathology arising from gene expression changes are due to mutant ATXN3 toxic gain-of-function mechanisms, rather than ATXN3 loss-of-function. In addition, we observed aberrant RNA splicing changes in KI mice, particularly in oligodendrocyte signature genes. Collectively, these novel findings position the spinal cord as a primary and early site of SCA3 pathogenesis and underscore its potential both as a sensitive regional biomarker for disease progression and as a key target for therapeutic intervention.

## 1 Introduction

Spinocerebellar ataxia type 3 (SCA3) is a debilitating and invariably fatal neurodegenerative disorder, currently with no disease-modifying treatments (Saute and Jardim, 2015; Paulson et al., 2017; Diallo et al., 2018; Matos et al., 2019; McLoughlin et al., 2020; Stahl et al., 2024). SCA3 is caused by a CAG repeat expansion in the *ATXN3* gene, resulting in an abnormally long polyglutamine (polyQ) tract in the ATXN3 protein (Kawaguchi et al., 1994; Watanabe et al., 1996). This mutant, polyQ-expanded, ATXN3 pathologically accumulates within vulnerable cell types, although the precise regional mechanisms leading to cellular dysfunction remain unknown (Paulson et al., 1997). Despite widespread expression of *ATXN3* throughout the body, neurodegeneration and protein accumulation in SCA3 predominantly impact select regions of the central nervous system (CNS), most notably the cerebellum and brainstem (Durr et al., 1996; Rüb et al., 2002a, 2002b; Guimarães et al., 2013; Koeppen, 2018; McLoughlin et al., 2020; Ferreira et al., 2024). These areas are well-recognized for their significant structural and functional decline in the disease, contributing to the classic motor and coordination deficits observed in patients (Guimarães et al., 2013; Kang et al., 2014; Wan et al., 2020; Ferreira et al., 2024; Ye et al., 2025). However, the spinal cord is also highly vulnerable in patients with SCA3, exhibiting substantial volumetric loss that can be detected by MRI prior to the onset of clinical symptoms and worsening as the disease advances (Lukas et al., 2008; Fahl et al., 2015; Rezende et al., 2018, 2024; Faber et al., 2021; Ye et al., 2025). Considering the early and progressive nature of spinal cord atrophy in SCA3 patients, it is well-positioned as a regional biomarker of SCA3. Yet, despite its clear degeneration and likely contribution to SCA3 patient symptoms, the spinal cord has often been overlooked in preclinical studies and therapeutic development, which tend to focus on the cerebellum and brainstem. The specific molecular alterations underlying spinal cord vulnerability in SCA3 remain largely unexplored, underscoring the need for a more comprehensive understanding to drive the development of more effective, targeted interventions.

To elucidate the molecular mechanisms underlying SCA3 pathogenic signatures in the spinal cord, we employed a transcriptomic approach across human patient samples and mouse models. We performed RNA sequencing on spinal cord samples from human SCA3 patients and controls to characterize gene expression changes associated with advanced disease. To capture the onset and progression of transcriptomic alterations, we analyzed spinal cord tissue from an SCA3 knock-in (KI) mouse model, which carries one wild-type and one hyperexpanded (300 CAG) *Atxn3* allele (Schuster et al., 2023; Putka et al., 2025), at both symptomatic onset (24 weeks) and end-stage disease (56 weeks). To complement this, we performed transcriptomic profiling in 24-week-old *Atxn3* knockout (KO) mouse spinal cord tissue to help distinguish between ATXN3 loss-of-function and toxic gain-of-function effects in SCA3 mice. We applied weighted gene correlation network analysis (WGCNA) and gene ontology (GO) enrichment analyses to identify biological pathways impacted by disease. In addition, we examined changes in RNA splicing across all datasets to further characterize molecular disruptions associated with SCA3. Altogether, this approach allowed us to systematically interrogate the transcriptional landscape of the SCA3 spinal cord in both human and mouse models, providing a novel foundation for future investigations interrogating molecular mechanisms driving spinal cord disease pathology.

## 2 Methods

### 2.1 Human samples

Human post-mortem frozen lumbar spinal cord tissue from SCA3 and control (cause of death not CNS-related) patients was acquired from the University of Maryland, Baltimore, and the University of Florida Center for NeuroGenetics biobanks. See Supplementary Table S1 for details on sample sex, age, postmortem interval, and ATXN3 CAG repeat size.

### 2.2 Mouse models

All KI experiments were conducted with heterozygous (*Atxn3*^Q300/Q6^) KI mice (Schuster et al., 2023; Putka et al., 2025; Ramani et al., 2017; RRID: IMSR_JAX:014603). KI mice in this study were generated by breeding a heterozygous female and wild type (*Atxn3*^Q6/Q6^) male mouse to limit further genetic anticipation. *Atxn3* KO (*Atxn3^−/−^*) mice used in these studies were first reported by Reina et al. (2012) and further characterized by our lab and others (Ramani et al., 2017; Zeng et al., 2018; Schuster et al., 2022a, 2022b; Putka et al., 2025). All mouse lines are maintained on a C57BL/6J background (RRID: IMSR_JAX:000664). See Supplementary Table S2 for details on the genotype, sex, and age of samples used in sequencing experiments. All animal procedures were approved by the University of Michigan Institutional Animal Care and Use Committee and conducted in accordance with the United States Public Health Service’s policy on Humane Care and Use of Laboratory Animals. Mice were housed in a room with standard 12-h light/dark cycles and food and water provided ad libitum.

### 2.3 Mouse genotyping

Mice were genotyped from tail DNA, biopsied before weaning for study enrollment, and confirmed upon postmortem tissue collection, as previously described (Schuster et al., 2023). KI mice were genotyped via PCR amplification of 80 ng of DNA using 10x PCR buffer, 5x Q Reagent, 10 mM DNTPs, and Taq from the Qiagen Taq PCR core kit per manufacturer’s instructions for a 25 μL reaction (Qiagen, 201223). Additionally, 4 μL of 5 M Betaine (Millipore Sigma, B0300), 1.6 μL of 10 μM forward and reverse primer, and ddH_2_O up to 25 μL were added per reaction. The PCR conditions were 95 °C for 2 min, followed by 40 cycles of 95 °C for 30 s, 55.2 °C for 1 min, and 72 °C for 2 min and 30 s, followed by a final step of 72 °C for 10 min. Primers flanking the endogenous mouse *Atxn3* CAG repeat (KI forward 5′-TTCACGTTTGAATGTTTCAGG-3′, KI reverse 5′-ATATGAAAGGGGTCCAGGTCG-3′) were used for this reaction. KO mice were genotyped via PCR amplification of 50 ng of DNA in a 25 μL reaction using 2x GoTaq Mix (Promega, M7832), 0.5 μL of each primer at 10 μM, and ddH_2_O up to 25 μL. The PCR conditions were 98 °C for 3 min, followed by 35 cycles of 96 °C for 30 s, 55 °C for 45 s, and 72 °C for 1 min, followed by a final step of 72 °C for 5 min. Primers used for KO mouse genotyping were: ATXN3KO forward 5′-GAGGGAAGTCGTCATAAGAGT-3′, ATXN3KO reverse 5′-TGGGCTACAAGAAATCCTGTC-3′, and ATXN3KO LTRa 5′-AAATGGCGTTACTTAAGCTAG-3′.

### 2.4 Tissue collection

Spinal cord tissue was collected at approximately 24 weeks of age from KI and KO mice (mean ± SEM = 24.08 ± 0.12 and 25.03 ± 0.17 weeks, respectively) and approximately 56 weeks of age from KI mice (56.86 ± 0.29 weeks). Mice were anesthetized with a lethal dose of ketamine-xylazine and then transcardially perfused with PBS before the spinal cord was collected via hydraulic expulsion (Richner et al., 2017). Cervical spinal cord tissue was flash frozen on dry ice for RNA experiments, and the remaining cord was post-fixed in 4% paraformaldehyde (PFA) for histology experiments.

### 2.5 RNA extraction and expression analysis

RNA was extracted from PBS-perfused, flash-frozen human and mouse spinal cord tissue, as previously described (Schuster et al., 2022b). Tissue was homogenized in RIPA buffer (Sigma Aldrich, R0278) with protease inhibitors (Sigma Aldrich, 11836170001) (1,000 μL for human samples and 500 μL for mouse samples) using a Next Advance Bullet Blender. Lysate was combined with Trizol (Invitrogen, 15596018), and RNA extraction was performed using QIAshredder (Qiagen, 79654) and RNeasy Plus Mini Kit (Qiagen, 74134), per the manufacturer’s protocol. RNA samples were submitted to the University of Michigan Advanced Genomics Core for library preparation and Illumina Next Generation Sequencing on a NovaSeq Flowcell. Mouse libraries were prepared using NEBNext Poly(A) mRNA Magnetic Isolation Module (NEB, E7490) and NEBNext UltraExpress RNA Library Prep Kit (NEB, E3330), while human samples were prepared using NEBNext rRNA Depletion Kit (NEB, E6310) and NEBNext UltraExpress RNA Library Prep Kit (NEB, E3330) due to RIN values less than seven (Supplementary Tables S1, S2).

FASTQ files provided by the University of Michigan Advanced Genomics Core underwent quality control using FastQC (version 0.12.1, RRID: SCR_014583) to ensure all samples used had sufficient read depth (>75 million paired-end reads) and check the adapter content (Supplementary Tables S1, S2). All FASTQ files had <20% adapter content, so no trimming was performed. FASTQ files were aligned to the GRCm39 mouse reference genome or GRCh38 human reference genome using STAR (version 2.7.10b, RRID: SCR_004463). Samtools (version 1.21, RRID: SCR_002105) was used to combine the dorsal and ventral spinal cord data from each patient. Differential gene expression was performed in RStudio (2025.05.0+513, RRID: SCR_000432; R version 4.4.2, RRID: SCR_001905) using DESeq2 (version 1.46.0, RRID: SCR_015687). Genes were considered differentially expressed if they passed a significance threshold of Padj < 0.05 and log2FC > |1.5|. Transcripts per million (TPM) values were calculated using Kallisto (version 0.46.2, RRID: SCR_016582). WGCNA was performed in RStudio using the WGCNA package (version 1.73, RRID: SCR_003302). g: Profiler [(Kolberg et al., 2023), RRID: SCR_006809] was used for all gene ontology (GO) analyses. Alternative splicing analysis was performed using rMATS (version 4.1.2, RRID: SCR_023485). Splicing events were considered significant if they passed a significance threshold of FDR < 0.05 and ΔPSI > |0.1|. All ΔPSI values are converted from a ratio to a percentage with a threshold-adjusted ΔPSI > |10%| accordingly. Exon numbers are defined by counting from the first exon in the ensemble transcript 201 or by matching coordinates to previous literature.

### 2.6 Immunohistochemistry

PBS perfused mouse spinal cords were post-fixed for 24 h in 4% PFA before being transferred into 30% sucrose in PBS for long-term storage at 4 °C. Spinal cords were dissected into 3 mm pieces, embedded in spinal racks (Fiederling et al., 2021) in OCT (Fisher, 4585) and cryostat-sectioned at 20 μm. For NeuN, GFAP, and IBA1 histology experiments, sections underwent a 30-min antigen retrieval in 0.01 M sodium citrate buffer (pH 8.5) at 80 °C, whereas ASPA histology experiments did not undergo an antigen retrieval step. Immunohistochemistry was performed using the Vectastain ABC-HRP Kit (Vector Laboratories, PK-4000) and DAB Substrate Kit (Vector Laboratories, SK-4100) per manufacturer’s protocol. Primary antibodies assessed include: rat anti-NeuN (1:500, Abcam, ab279297, RRID: AB_3095692), rabbit anti-ASPA (1:500, Millipore Sigma, ABN1698, RRID: AB_2827931), mouse anti-GFAP (1:500, Cell Signaling Technology, 3,670 s, RRID: AB_561049), and rabbit anti-IBA1 (1:500, Fisher, PIPA521274, RRID: AB_2804971). Coverslips were mounted using DPX mounting media (Electron Microscopy Science, 13510), and imaging was performed using a Nikon Ti2 widefield microscope (RRID: SCR_021068) with a Digital Sight 10 camera.

### 2.7 Statistics

Statistical analyses were carried out in Microsoft Excel, GraphPad Prism (10.4.1), and RStudio (2025.05.0+513). The PCA plots and WGCNA dendrogram were created in RStudio using ggplot2 and WGCNA. Ellipses on PCA plots represent 95% confidence intervals. Graphs display the mean ± SEM with significance levels as follows: ns = not significant, * *p* < 0.05, ** *p* < 0.01, *** *p* < 0.001, or **** *p* < 0.0001.

## 3 Results

### 3.1 Transcriptomic profiling reveals widespread gene dysregulation in human SCA3 spinal cord

Although MRI and post-mortem studies have established pronounced structural changes and atrophy in the spinal cord of SCA3 patients (Pinto and De Carvalho, 2008; Suga et al., 2014; Pedroso et al., 2016). The molecular mechanisms underlying this vulnerability remain poorly defined, and no transcriptional analyses have been reported to date. To address this gap, we performed bulk RNA sequencing on spinal cord samples collected post-mortem from four SCA3 patients and four age-matched controls (non-CNS-related cause of death) (Supplementary Table S1). RNA was extracted from frozen spinal cord samples received from the University of Florida and the University of Maryland, Baltimore Brain Banks. Due to suboptimal RNA quality (RIN < 7), samples were prepared with ribosomal RNA depletion followed by paired-end sequencing, yielding an average of 93.6 million reads per sample (standard deviation 20.4 million) (GSE309548).

**Figure 1:**
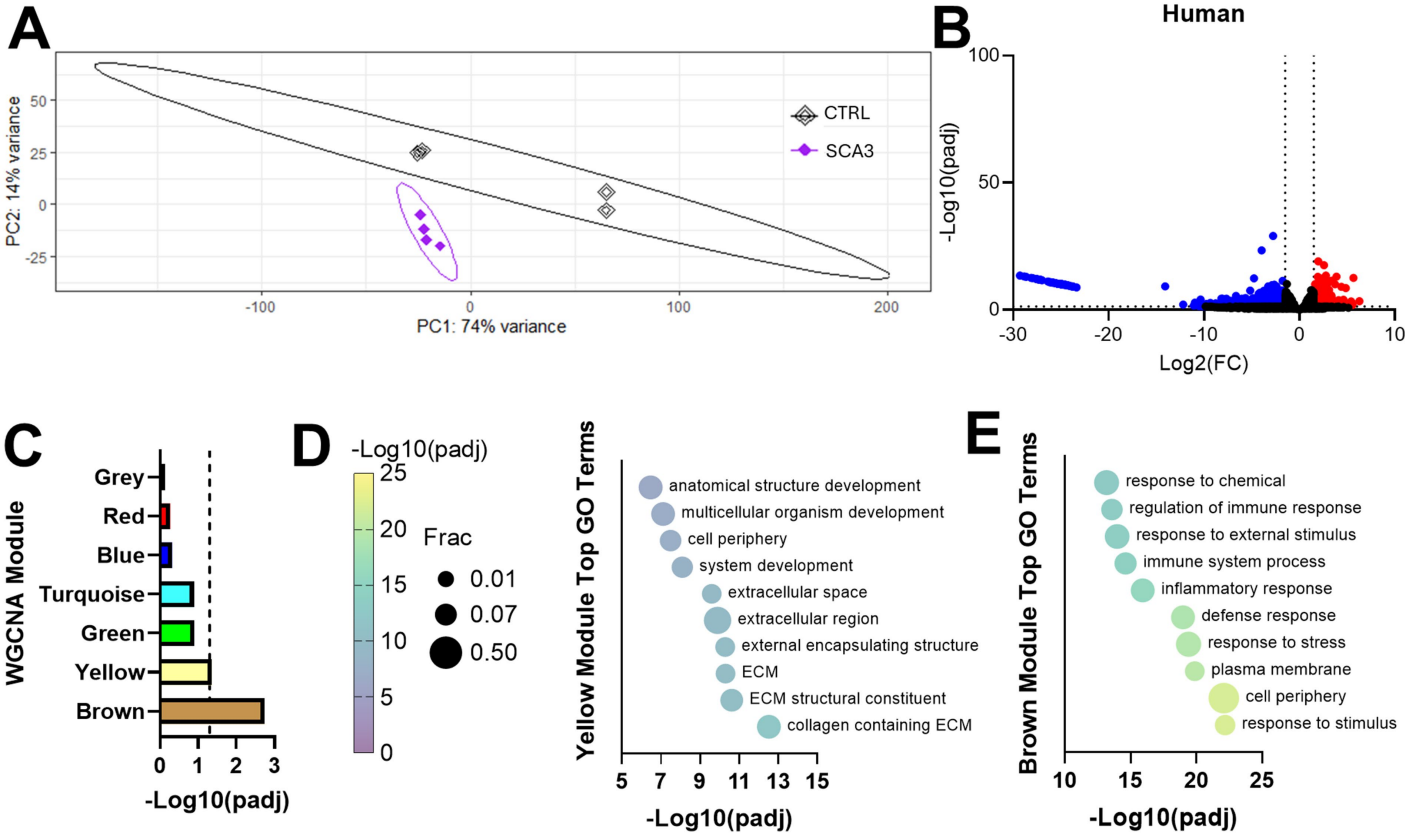
Human post-mortem SCA3 tissue shows significant transcriptional dysregulation. **(A)** PCA plot of gene expression differences from human SCA3 and control (CTRL) spinal cord samples, with ellipses representing 95% confidence intervals for group. **(B)** Volcano plot highlighting significantly upregulated (red) and downregulated (blue) differentially expressed genes between SCA3 and CTRL samples. **(C)** Significant disease-associated weighted gene co-expression network analysis (WGCNA) modules. **(D,E)** Top 10 most significant gene ontology (GO) terms for **(D)** brown and **(E)** yellow WGCNA modules. Frac depicts the fraction of GO term genes included in the dataset.

Principal component analysis (PCA) demonstrated clear separation between SCA3 and control samples (Figure 1A). Differential gene expression analysis revealed widespread transcriptional dysregulation in the SCA3 spinal cord, with 466 upregulated and 1,189 downregulated differentially expressed genes (DEGs) (Figure 1B; Supplementary Table S3). To further interpret these changes, we applied weighted gene co-expression network analysis (WGCNA) to detect groups of co-expressed, functionally related gene modules (Supplementary Figure S1; Supplementary Table S4). Of these, two modules—brown and yellow—were significantly altered in SCA3 samples compared to controls (Figure 1C). Gene ontology (GO) analysis revealed that the brown module was enriched for genes involved in inflammation, while the yellow module was associated with extracellular matrix (ECM) organization (Figures 1D,E; Supplementary Table S5). These results align with previous reports of neuroimmune activation and ECM remodeling in SCA3-vulnerable brain regions, including the cerebellum and brainstem (Evert et al., 2001; Rüb et al., 2002a, 2002b; Scherzed et al., 2012; Liu et al., 2025). The ECM has previously been implicated in SCA3 disease pathology and may contribute to the structural changes observed in the patient’s spinal cord by MRI (Rezende et al., 2018, 2024; Faber et al., 2021; Ye et al., 2025). Although analysis of human end-stage tissue has highlighted key dysregulated pathways in the SCA3 spinal cord, it does not clarify when these changes arise or how they progress during disease, prompting us to investigate these questions using SCA3 mouse models.

### 3.2 Spinal cord transcriptional dysregulation is early and progressive in a SCA3 KI mouse model

To overcome the limitations of end-stage human tissue, we used our KI SCA3 mouse model, which expresses one normal copy of *Atxn3* with ~6 CAG repeats and one disease copy of *Atxn3* with ~300 CAG repeats (Schuster et al., 2023; Putka et al., 2025). These mice develop motor symptoms beginning around 24 weeks of age and typically reach end-stage by 56 weeks (Putka et al., 2025). We collected spinal cord RNA at both these timepoints to assess early and progressive transcriptomic changes. RIN values for all samples were greater than or equal to 8.5, allowing for polyA library prep and paired-end sequencing that resulted in an average read count of 96.7 million (standard deviation 7.5 million) at 24 weeks and 80.2 million (standard deviation 8.3 million) at 56 weeks (GSE309535).

**Figure 2:**
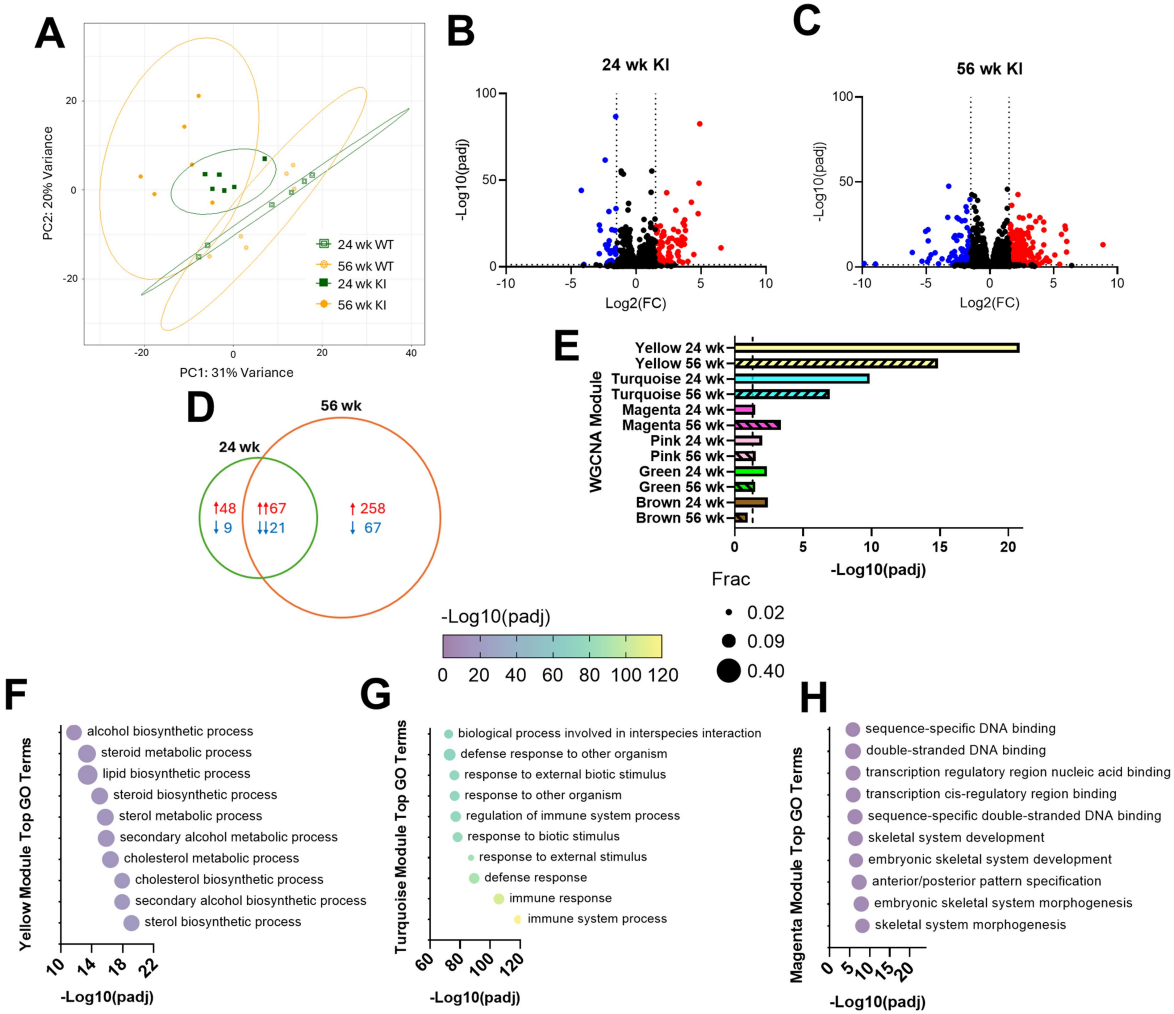
Transcriptional dysregulation occurs early and progressively in the spinal cord of SCA3 KIQ300 mice. **(A)** PCA plot of early (24 week) and late (56 week) stage gene expression differences from KI and WT littermate controls, with ellipses representing 95% confidence intervals. **(B,C)** Volcano plots highlight significantly upregulated (red) and downregulated (blue) differentially expressed genes in **(B)** 24- and **(C)** 56-week samples. **(D)** Venn diagram of overlapping differentially expressed genes in 24- and 56-week spinal cord. **(E)** Significant disease-associated weighted gene co-expression network analysis (WGCNA) modules in 24- and 56-week samples. **(F–H)** Top 10 most significant gene ontology (GO) terms for **(F)** yellow, **(G)** turquoise, and **(H)** magenta WGCNA modules. Frac depicts the fraction of GO term genes included in the dataset.

PCA plots demonstrated that 24- and 56-week SCA3 KI mice progressively diverge from age-matched wild type (WT) controls, consistent with early and advancing transcriptomic changes (Figure 2A). Differential gene expression analysis identified 145 DEGs at 24 weeks and 413 DEGs at 56 weeks (Figures 2B,C; Supplementary Table S3). The majority of the 24-week DEGs remain differentially expressed in the same direction at 56 weeks (Figure 2D), supporting progressive transcriptional dysregulation in the SCA3 spinal cord. Unbiased WGCNA revealed six significantly dysregulated gene modules in the KI spinal cord (Figure 2E; Supplementary Figure S1; Supplementary Table S4). Of particular note, the yellow and turquoise modules were consistently significant at both 24- and 56-week timepoints. Yellow module genes function in lipid metabolism pathways (Figure 2F; Supplementary Table S5), a group of biological functions previously found to be affected by SCA3 in vulnerable brain regions (Schuster et al., 2022b, 2023; Putka et al., 2025), while the turquoise module represented inflammatory pathways (Figure 2G; Supplementary Table S5) similar to that seen histologically in SCA3 patient and mouse model pontine tissue (Evert et al., 2001; McLoughlin et al., 2018). Additionally, the magenta module demonstrated progressive changes and was enriched for terms related to nucleic acid processing and cell development (Figure 2H; Supplementary Table S5), echoing recent findings that link RNA splicing disturbances to SCA3 pathogenesis (Shorrock et al., 2023; Liu et al., 2025).

### 3.3 *Atxn3* knockout spinal cord shows a transcriptional profile distinct from SCA3

To determine the contributions of ATXN3 loss of function to the transcriptomic changes observed in KI mice, we performed RNA sequencing on spinal cords from 24-week *Atxn3* KO mice and their WT littermate controls. All samples had RIN values greater than or equal to 8.9, indicating sufficient quality for polyA library preparation. Sequencing yielded an average of 115 million paired-end reads per sample (standard deviation 12 million) (GSE309549).

**Figure 3:**
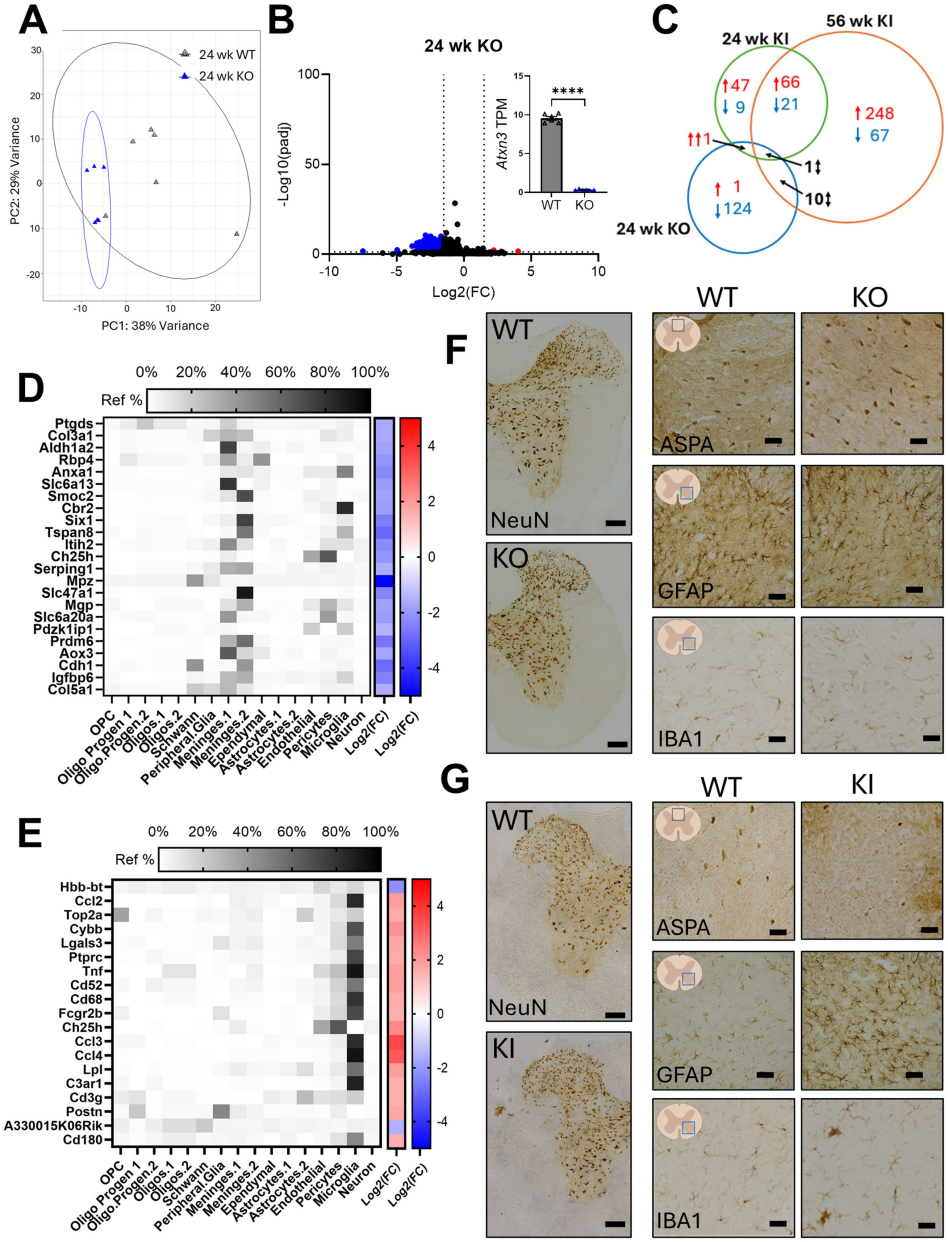
*Atxn3* knockout (KO) mouse spinal cords display a distinct transcriptional profile relative to SCA3 KIQ300 mouse spinal cords. **(A)** PCA plot of gene expression data from 24 week KO and WT littermate controls, with ellipses representing 95% confidence intervals. **(B)** Volcano plot highlighting upregulated (red) and downregulated (blue) differentially expressed genes (DEGs) in KO samples. Inset bar graph confirms knockdown of *Atxn3* transcripts in KO samples relative to controls. **(C)** Venn diagram shows overlapping DEGs in 24 week KO, 24 and 56 week KIQ300 spinal cord. **(D)** Heatmap showing cell enrichment and log_2_ fold change of *Atxn3* KO DEGs from cell type expression is based on single-nucleus RNA sequencing of mouse spinal cord tissues (Russ et al., 2021). **(E)** Heatmap showing cell enrichment and log_2_ fold change of spinal cord cell type expression data for 24 week KI DEGs (Russ et al., 2021). **(F)** Immunohistochemical analysis shows major CNS cell type markers in 24 week *Atxn3* KO mouse spinal cord: ASPA representative image from dorsal column. GFAP and IBA1 representative images from the ventral horn. **(G)** Immunohistochemical analysis of major CNS cell type markers in 24 week SCA3 KI mouse spinal cord, as in **(E)** (Immunohistochemical studies completed in *n* = 3 mice/genotype, raw images in DANDI archive, DANDI set 001620, NeuN scale bar = 100 μm, all others scale bar = 50 μm).

PCA showed distinct clustering of the KO mice relative to WT controls (Figure 3A). Differential gene expression analysis revealed substantially fewer DEGs in the KO spinal cord relative to KI mice, with almost all DEGs downregulated (Figure 3B; Supplementary Table S3). Comparing the 138 KO DEGs to those in the SCA3 KI datasets showed minimal overlap; only one of twelve shared genes (*Or5v1b*) was dysregulated in the same direction, and notably, *Or5v1b* has no known connection to SCA3 disease (Figure 3C). To identify major dysregulated cell types, we utilized a published mouse spinal cord atlas (Russ et al., 2021) and found no specific cell types overrepresented among KO DEGs (Figure 3D). In contrast, analysis of the 24-week KI tissue using the same atlas revealed enrichment of microglial genes among DEGs (Figure 3E), a pattern also observed in the 56-week KI data set (Supplementary Figure S2). Importantly, the spinal cord atlas was derived from healthy mice, where astrocytes are quiescent and have gene profiles distinct from activated cells. As a result, transcriptomic analyses using this reference may underestimate reactive astrocyte signatures. Peroxidase-based immunohistochemistry of major CNS cell type markers in 24-week KO spinal cord confirmed the lack of remarkable changes in neurons, oligodendrocytes, astrocytes, or microglia (Figure 3F). However, in the 24-week KI spinal cord, histological analysis showed decreased expression of a marker for mature oligodendrocytes (ASPA) and increased markers for reactive astrocytes (GFAP) and microglia (IBA1) (Figure 3G). The affected cell types in the SCA3 spinal cord correspond to those previously reported in vulnerable brain regions and are not dysregulated in the absence of ATXN3 (Evert et al., 2001; Ramani et al., 2017; McLoughlin et al., 2018; Duarte Lobo et al., 2020; Schuster et al., 2022b, 2023).

### 3.4 RNA splicing is dysregulated in the SCA3 spinal cord

**Figure 4:**
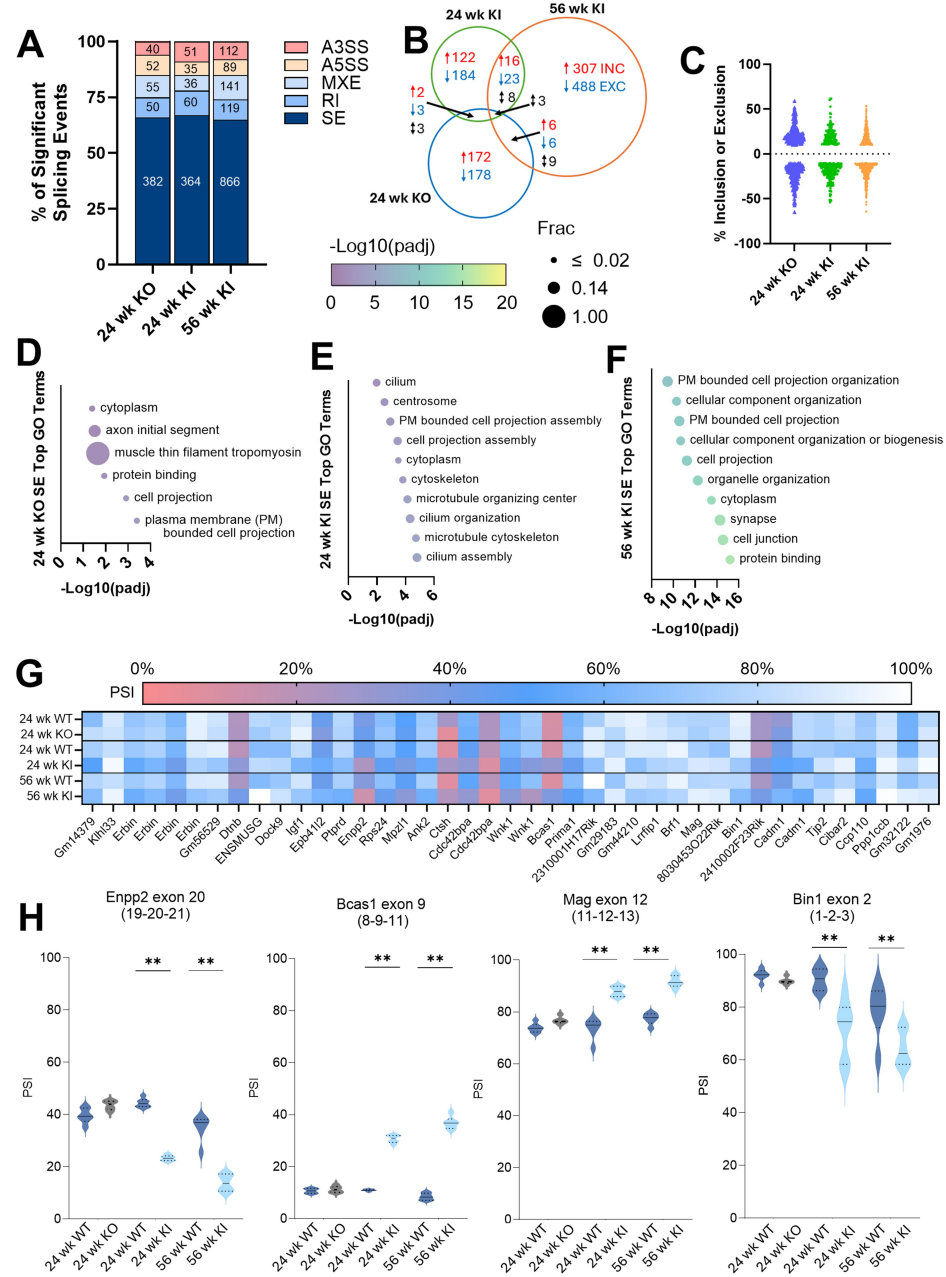
RNA splicing is dysregulated in the spinal cord of SCA3 mouse models. **(A)** Percentage of significantly mis-spliced skipped exon (SE), retained intron (RI), mutually exclusive exons (MXE), alternative 5′ splice site (A5SS), and alternative 3′ splice site (A3SS) events as a proportion of all significant splicing events in 24-week KO, 24-week KI, and 56-week KI spinal cord datasets, FDR < 0.05, ΔPSI > 10%. **(B)** Venn diagram of SE events that overlap, at all six coordinates, between datasets. **(C)** Percentage of exon inclusion (positive) or exclusion (negative) for significant SE events in each dataset, FDR < 0.05, ΔPSI > 10%. **(D)** Top 10 gene ontology (GO) terms derived from *Atxn3* KO mouse SE events. **(E)** Top 10 GO terms derived from 24-week KI SE events. **(F)** GO terms derived from 56-week KI SE events. **(G)** Heatmap showing average percent spliced-in (PSI) of SE events shared between 24- and 56-week KI datasets, but not in the 24-week KO dataset. Frac depicts the fraction of GO term genes included in the dataset. **(H)** Violin plots of select oligodendrocyte-related genes with significant SE dysregulated in KI mice. Parentheses denote (upstream exon-skipped exon-downstream exon).

Recent studies have uncovered significant RNA splicing dysregulation in SCA3, particularly affecting genes involved in synaptic signaling and the cytoskeleton (Shorrock et al., 2023; Aliyeva et al., 2025; Lauerer et al., 2025; Liu et al., 2025). To determine if similar splicing alterations occur in our spinal cord datasets, we systematically analyzed five types of alternative splicing events, alternative 3′ start site (A3SS), alternative 5′ start site (A5SS), mutually exclusive exons (MXE), retained intron (RI), and skipped exon (SE), setting significance cutoffs of FDR < 0.05 and |PSI| > 0.1. The 56-week SCA3 KI spinal cord samples exhibited more significant alternative splicing events than the 24-week KI and KO samples, with SE events comprising the majority in all data sets (Figure 4A). KO mice showed slightly more significant splicing events than the 24-week KI mice (Figure 4A), but there was very little overlap between significant splicing events between models (Figure 4B). Given that SE events dominated the splicing changes and mirrored findings from prior SCA3 studies, we further characterized these events. In all datasets, SE events showed roughly equal distribution between exon inclusion and exclusion when compared to WT controls (Figure 4C; Supplementary Table S6). GO analysis of SE events in mouse and human datasets consistently highlighted enrichment of terms related to cytoskeletal structure and cell projections (Figures 4D–F; Supplementary Figure S3; Supplementary Table S7). This overlap in SE-derived GO terms between KI and KO datasets was surprising to us, especially considering the minimal number of shared events in these datasets. We hypothesize this is because GO uses genes as inputs rather than skipped exon events, and of the unique events in the KI and KO datasets, there is a large portion of commonly affected genes. So, while these genes are affected differently at the splicing level in KI and KO tissue, they are contributing to the GO analysis in the same way. This is a limitation of using GO with alternative splicing data, and so we moved to look at the SE events shared between our KI datasets. In plotting the events that were dysregulated in the same direction in the 24- and 56-week KI datasets (Figure 4G), we made two key observations. First, many KI SE events, such as *Bcas1*, *Itpr1*, and *Kcnma1*, were previously found to be early and progressively dysregulated in the cerebellum of two other mouse models of SCA3 and a mouse model of SCA1 (Shorrock et al., 2023). Additionally, several events shared across our KI datasets are related to oligodendrocytes (Figure 4H). The first gene, *Enpp2*, is a marker of mature, myelinating oligodendrocytes and has been implicated in several neurodegenerative disorders (Aston et al., 2005; Yuelling et al., 2012). *Bcas1*, previously found to be dysregulated in the cerebellum of SCA3 mice, is associated with specific populations of myelinating oligodendrocytes in the context of Multiple Sclerosis (Fard et al., 2017; Ishimoto et al., 2017; Shorrock et al., 2023). *Mag* encodes a key structural myelin component, and splicing changes or mutations in Mag have been linked to myelination deficits and ataxic symptoms (Wu et al., 2002; Roda et al., 2016; Santos et al., 2020; Zech et al., 2020). *Bin1* splicing has also been associated with changes in oligodendrocyte function in Alzheimer’s Disease (De Rossi et al., 2016). Overall, the persistence of splicing changes in these genes connected to common pathological mechanisms in SCA3 suggests that splicing may be an important driver of disease and warrants further investigation.

## 4 Discussion

Despite decades of research focused on cerebellar degeneration in SCA3, the spinal cord has now emerged as another key driver of early disease processes and symptom progression, yet its underlying molecular pathology remains largely unexplored (Rezende et al., 2018, 2024; Faber et al., 2021; Ye et al., 2025). Our study offers the first in-depth transcriptional interrogation of this vulnerable region, revealing a complex interplay of early and progressive gene expression and splicing disruptions that extend beyond the brainstem and cerebellar regions typically associated with SCA3. We established toxic gain-of-function transcriptional signatures and pathology in the SCA3 spinal cord consistent with previous reports in other brain regions, providing groundwork for future mechanistic and therapeutic studies (Schmitt et al., 2007; Reina et al., 2012; Ramani et al., 2017; Schuster et al., 2022a, 2022b, 2024; Putka et al., 2025).

A notable finding from both human SCA3 and KI mouse data is that transcriptional dysregulation in the spinal cord involves great changes to lipid metabolism, inflammation, and extracellular matrix composition. These processes are increasingly recognized as central players in neurodegeneration. Importantly, they mirror pathological mechanisms previously established in other SCA3-affected brain regions, including changes in oligodendrocyte maturation (Schuster et al., 2022b, 2023), inflammation (Evert et al., 2001; Rüb et al., 2002a, 2002b; Scherzed et al., 2012), cytoskeletal function (Neves-Carvalho et al., 2015; Wiatr et al., 2019, 2021), and nucleic acid biology (Kazachkova et al., 2013; Chatterjee et al., 2015). Previous work from our group showed that altered lipid metabolism and impaired oligodendrocyte maturation begin prior to symptom onset in the brain, and now, as supported by the current data, in the spinal cord as well (Schuster et al., 2022b, 2023; Putka et al., 2025). Evidence of downregulation of mature oligodendrocyte markers, along with disrupted lipid metabolism genes, supports a spinal cord component to white matter pathology in SCA3. These molecular changes may underlie the early and robust atrophy seen in imaging studies of the patient’s spinal cord (Rezende et al., 2018, 2024; Faber et al., 2021; Ye et al., 2025) and provide concrete mechanistic links between cellular dysfunction and structural neurodegeneration.

Previous studies in SCA3 vulnerable brain regions, including the brainstem and pons, have documented astrocytic and microglial reactivity similar to our spinal cord findings (Evert et al., 2001; Rüb et al., 2002a, 2002b; Scherzed et al., 2012). However, the inflammatory spinal cord signatures were prevalent even at early symptomatic stages in this KI mouse model. This challenges the prevailing view that neuroinflammation is merely a late or reactive pathology, suggesting instead that it may have a more active role in SCA3 dysfunction within the spinal cord. Whether this inflammation is a cause or consequence of glial and neuronal dysregulation remains unclear; however, our data highlight it as an important target for future mechanistic and therapeutic studies.

Our transcriptomic analyses also uncovered significant splicing alterations, especially in genes governing cytoskeletal structure and myelination. These results align with recent studies implicating aberrant RNA processing as a cross-disease mechanism in polyglutamine disorders and expand it to the spinal cord in SCA3 mouse and human tissue (Daughters et al., 2009; Mykowska et al., 2011; Li et al., 2016; Lin et al., 2016; Schilling et al., 2019; Elorza et al., 2021; Olmos et al., 2023; Shorrock et al., 2023; Aliyeva et al., 2025). The observed dysregulation of *Bcas1*, *Mag*, *Bin1*, and *Enpp2* splicing, genes central to myelination by oligodendrocytes (Wu et al., 2002; Yuelling et al., 2012; De Rossi et al., 2016; Fard et al., 2017; Ishimoto et al., 2017), provides a tantalizing molecular link between RNA splicing defects and impaired myelin maintenance in SCA3 (Schuster et al., 2022b, 2023). It remains to be seen whether these splicing changes precede, drive, or result from other pathological alterations in the diseased spinal cord.

Our findings suggest that the spinal cord is not simply a downstream casualty of SCA3 brain pathology, but rather another primary site of early and progressive molecular dysfunction. The fact that spinal cord atrophy emerges before overt ataxia in patients by MRI and closely tracks with disease severity, highlights its strong potential as a biomarker for monitoring disease progression and evaluating therapeutic responses. At the same time, the early and multifaceted nature of molecular disruption in the spinal cord, encompassing transcriptomic, splicing, metabolic, and inflammatory changes, raises pivotal questions about which cell types are most critical in disease onset, whether these molecular alterations represent parallel or interconnected pathogenic pathways, and importantly, whether such changes can be targeted or reversed to alter SCA3 progression. In summary, our study is the first to position the spinal cord as a central player in SCA3 pathogenesis with both commonalities and distinctions from other vulnerable regions, and by identifying fundamental molecular changes at early disease stages, provides an essential framework for future research to decipher pathogenic mechanisms and ultimately guide the development of targeted, effective therapies for this devastating disorder.

## Data Availability

The datasets presented in this study can be found in online repositories. The names of the repository/repositories and accession number(s) can be found in the article/Supplementary material.
