# Supplementary material for: Early transcriptomic perturbations highlight the spinal cord as a key pathogenic region in spinocerebellar ataxia type 3
Source: Front Cell Neurosci. 2026 Jan 14;19:1735225. doi: 10.3389/fncel.2025.1735225 (PMC12846992; doi:10.3389/fncel.2025.1735225)
Supplement: Supplementary file 1 [file Data_Sheet_1.PDF]

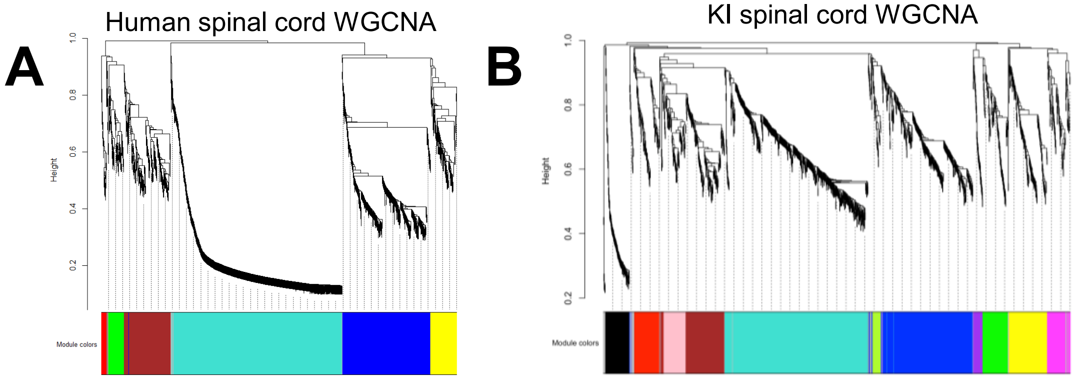

Figure S1. Dendrograms illustrate module breakdown in Human and KI spinal cord sequencing data. **A)** Dendrogram derived from human spinal cord bulk RNA sequencing data. **B)** Dendrogram derived from 24- and 56-week KI mouse spinal cord sequencing data.

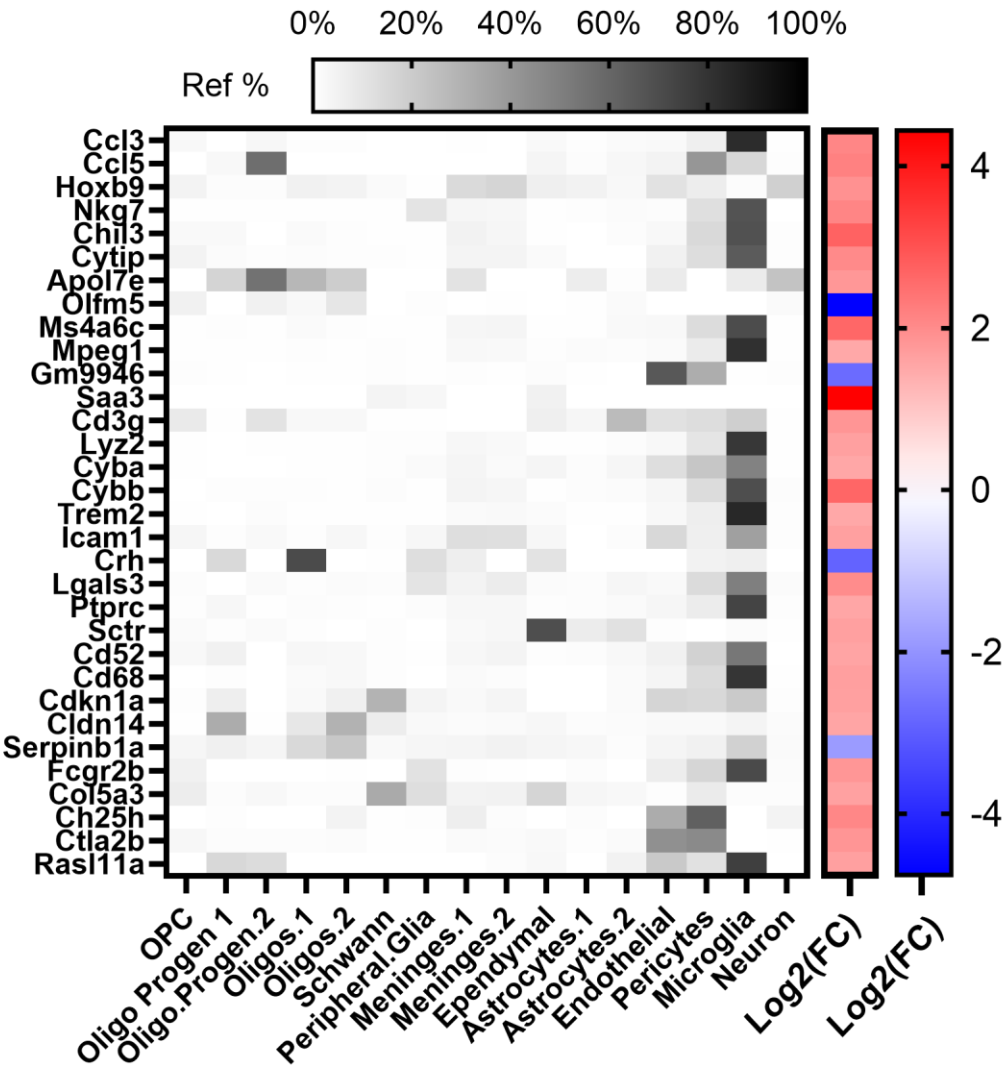

Figure S2. Differentially expressed genes in the 56-week SCA3 KI mouse spinal cord are enriched in microglial populations. Heatmap displays cell enrichment and log2fold change of 32 56-week KI DEGs (based on publicly-available data from Russ et al. 2021).

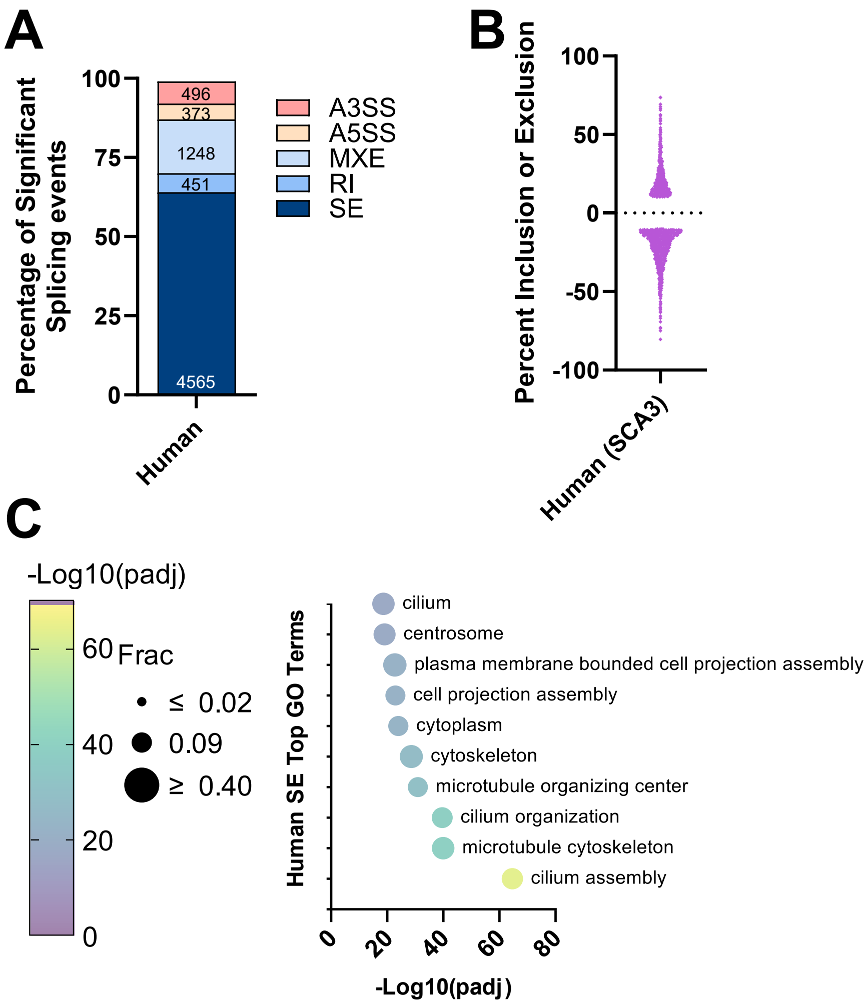

Figure S3. Alternative splicing is dysregulated in human SCA3 spinal cord tissue. **A)** Graph showing significant splicing events in human SCA3 spinal cord tissue. **B)** Distribution of inclusion and exclusion skipped exon events. **C)** Top 10 GO terms derived from human SE events. Frac depicts the fraction of GO term genes included in the dataset.
